# Development and validation of the screening tool for age-related hearing loss in the community based on the information platform

**DOI:** 10.1007/s00405-023-08389-9

**Published:** 2024-01-11

**Authors:** Jianli Ge, Yunyun Yan, Yinqian Zhu, Xin Cheng, Huazhang Li, Xiaoming Sun, Hua Jiang

**Affiliations:** 1 Department of General Practice, Shanghai East Hospital, Tongji University School of Medicine, Shanghai, 200120 China; 2 Department of General Practice, Huashan Hospital, Fudan University, Shanghai, 200040 China; 3 Department of Science and Education, Shanghai Guangming Traditional Chinese Medicine Hospital, Shanghai, 201399 China; 4 Department of General Practice, Zhongshan Hospital, Fudan University, Shanghai, 200032 China

**Keywords:** Age-related hearing loss, Screening software, Community, General practitioner, Loop management

## Abstract

**Introduction:**

Currently, age-related hearing loss has become prevalent, awareness and screening rates remain dismally low. Duing to several barriers, as time, personnel training and equipment costs, available hearing screening tools do not adequately meet the need for large-scale hearing detection in community-dwelling older adults. Therefore, an accurate, convenient, and inexpensive hearing screening tool is needed to detect hearing loss, intervene early and reduce the negative consequences and burden of untreated hearing loss on individuals, families and society.

**Objectives:**

The study harnessed "medical big data" and "intelligent medical management" to develop a multi-dimensional screening tool of age-related hearing loss based on WeChat platform.

**Methods:**

The assessment of risk factors was carried out by cross-sectional survey, logistic regression model and receiver operating characteristic (ROC) curve analysis. Combining risk factor assessment, Hearing handicap inventory for the elderly screening version and analog audiometry, the screening software was been developed by JavaScript language and been evaluated and verified.

**Results:**

A total of 401 older adults were included in the cross-sectional study. Logistic regression model (univariate, multivariate) and reference to literature mention rate of risk factors, 18 variables (male, overweight/obesity, living alone, widowed/divorced, history of noise, family history of deafness, non-light diet, no exercising habit, smoking, drinking, headset wearer habit, hypertension, diabetes, hyperlipidemia, cardiovascular and cerebrovascular diseases, hyperuricemia, hypothyroidism, history of ototoxic drug use) were defined as risk factors. The area under the ROC curve (AUC) of the cumulative score of risk factors for early prediction of age-related hearing loss was 0.777 [95% CI (0.721, 0.833)]. The cumulative score threshold of risk factors was defined as 4, to classify the older adults into low-risk (< 4) and high-risk (≥ 4) hearing loss groups. The sensitivity, specificity, positive predictive value, and negative predictive value of the screen tool were 100%, 65.5%, 71.8%, and 100.0%, respectively. The Kappa index was 0.6.

**Conclusions:**

The screening software enabled the closed loop management of real-time data transmission, early warning, management, whole process supervision of the hearing loss and improve self-health belief in it. The software has huge prospects for application as a screening approach for age-related hearing loss.

**Supplementary Information:**

The online version contains supplementary material available at 10.1007/s00405-023-08389-9.

## Introduction

### Age-related hearing loss

Age-related hearing loss (ARHL) is defined as hearing loss caused by aging and degeneration of auditory organs. The main risk factors include aging, environmental noise, smoking, drinking, genetic susceptibility, diabetes, hypertension, hyperuricemia, hyperlipidemia, hypothyroidism, ototoxic drugs, ear inflammation, etc. Although the pathogenesis of ARHL remains unclear it is widely thought to result from the joint action of multiple physiological mechanisms [1–3].

During early stages, the person's symptoms are binaural symmetrical high-frequency hearing loss, which can be accompanied by tinnitus in some cases, resulting in the decline of auditory recognition ability. However, the early subjective sensory speech recognition ability can meet daily communication needs, and the symptoms are easily ignored.

Current evidence suggests that hearing loss affects 33%, 45% and 63.1% of persons over 50, 60 [4]and 70 [5], respectively. Old adults with hearing loss are at significantly greater risk of incident dementia [5–7], falls [8, 9], depression [10, 11], social isolation [12], and loss of independence [13]. The latest projections suggest that hearing loss will be the 9th leading contributor to the global burden of disease worldwide in 2030 [14]. The medical costs of hearing loss range from $3.3 to $12.8 billion in the United States [15, 16] and $11.75 billion in Australia [17]. The serious adverse consequences of ARHL are often largely underestimated by society, older adults, healthcare professionals, and superintendents [18, 19]. It has been established that treating hearing loss may effectively reduce the adverse consequences. Nevertheless, the key to early treatment is identifying individuals with hearing loss as soon as possible [20, 21].

### Screening of age-related hearing loss

The United States Centers for Disease Control and Prevention (CDC) reported that 97% of newborn babies underwent hearing screening in 2013. In China, the rate of newborn hearing screening reached more than 60% in 2021, and the completion rate in Shanghai was 100%. However, the screening of ARHL is still in its infancy, and there is no unified view of the data [22–26].

Globally, compared with developed countries (10–40%), less than 1% of hearing loss patients in developing countries receive hearing aid treatment. Although improvements have been made to treating hearing loss, the acceptance and use rates of hearing aids have not increased significantly in the past 50 years [27]. More than 95% of persons who can benefit from hearing aids do not use hearing aids. Currently, the cognition and willingness to seek the help of older adults are very low. Little emphasis has hitherto been placed on ARHL screening, with no effective referral management means available.

One of the gold standard for assessing hearing loss is pure tone audiometry (PTA); however, this method is not feasible for large-scale, population-based epidemiological screening projects, because it requires high-cost audiological equipment and trained specialists [28]. The other screening methods of ARHL include the subjective faces scale [29], whispering experiment ^31^, hearing handicap inventory for the elderly (HHIE) [31], and screening for ontological functional impairments (SOFI) [32]. It has been shown that despite the advent of screening tools for hearing loss, they exhibit limitations in sensitivity, specificity, positive predictive value, and negative predictive value [24]. Screening of hearing loss should be weighed according to the sensitivity, specificity, technological requirements, per capita labor cost and ease of operation to improve the effectiveness of screening and reduce ineffective referral.

### Intelligent medicine and chronic disease

Smart medicine originated from the concept of smart earth and was put forward by Peng Mingsheng of IBM (International Business Machines Corporation) in the United States in November 2008. This project aimed to achieve medical information interconnection, medical technological innovation, scientific evidence-based diagnosis and big data of public health prevention and management through the Internet of Things technology from the perspective of information technology [33].

Intelligent medicine represents a driving force for innovation in treating incurable diseases. With the emergence of various wearable medical devices, we can timely obtain the information indicators of various monitoring data fed back by medical devices to prevent and control the development of diseases. The application of big data and artificial intelligence enables us to obtain comprehensive information, understand medical records and even the details of medical processes, and harness high-speed logical operation to process and analyze problems in medical treatment and corresponding management. With the emergence of smartphones, various mobile health applications have been developed, providing strong help for managing chronic disease and improving health-related status [34].

A systematic review and meta-analysis on the intervention effect of hypertensive patients based on smartphone applications, which included eight studies involving 1657 subjects, suggested that smartphone program intervention could reduce the blood pressure of hypertensive patients and increase drug compliance [26]. A randomized controlled feasibility study showed that mHealth could help persons with COPD self-manage their physical activity levels [35].

The occurrence of ARHL is related to many behavioral factors and disease factors. Although the primary prevention of ARHL cannot be accurately implemented, we can refer to the management policy of chronic diseases to implement secondary prevention and tertiary prevention.

## Materials and methods

### Assessment of risk factors of ARHL

#### Participants

Five community health centers in Pudong New Area, Shanghai [urban (*n* = 3), urban–rural fringe (*n* = 1) and suburban (*n* = 1)] were selected by the stratified sampling method. Older adults who participated in the annual physical examination (National public health service project) in these community health service centers from January to December 2019 were included in the present study.

#### Data collection

1) General information:

The participants were asked to complete a questionnaire, including the following contents:

Socio-demographic variables (age, gender, residential area, whether living alone,, marital status, education background), lifestyle habits (history of smoking, history of drinking, exercising habit, diet, headphone-wearing), physiological variables (weight, height, BMI, blood pressure), history of chronic disease (hypertension, diabetes, cardiovascular disease, hyperlipidemia, hyperuricemia, history of chronic otitis media, history of noise exposure, family history of deaf, history of ototoxic drug use).

2) Sample size calculation:

According to Kendall's sample estimation method, the number of observations is at least 10 times the number of variables. The indicators involved in this project include 7 sociodemographic statistical variables, 5 physiological factor indicators, and 14 hearing assessments, totaling 26 statistical analysis variables. Approximately 260 older adults, considering invalid questionnaires and no response rates, assuming an effective sample recovery rate of 70%, the minimum sample size is 371.

3) Hearing handicap inventory for the elderly screening version (HHIE-s):

HHIE-s consists of 10 items involving 5 emotional and 5 situational problems with three options: yes, sometimes, never. The corresponding scores are 4, 2 and 0, respectively. The lowest score is 0, and the highest score is 40. The scale was completed within 5 min, with higher scores associated with more serious hearing loss. According to the American Speech–Language–Hearing Association hearing screening guidelines, 0–8, 10–22, and 24–40 points correspond to no obvious, mild to moderate, and severe hearing impairment, respectively [3].

4) Audiometric assessment:

Puretone audiometry (PTA) is a commonly used method for hearing monitoring. Audiometric pure tone testing was administered by two trained audiologists. The Madsen audiometer was used in the room, where the indoor noise was controlled within 40 dB. Air conduction data were conducted, and the average value of 0.5 kHz, 1 kHz, 2 kHz and 4 kHz pure tone air conduction threshold of the better ear was taken as audiometric results. ≤ 25 dB/HL, 26–40 dB/HL, 41–55 dB/HL, 56–70 dB/HL,71–90 dB/HL and ≥ 91 dB/HL correspond to normal, mild hearing loss, moderate hearing loss, moderately severe hearing loss, severe hearing loss and profound hearing loss, respectively [20, 36, 37].

5) Assessment of risk factors:

Based on the cross-sectional data, using conditional logic equations, assign values of 1 or 0 to whether they have risk factors, and obtain the cumulative scores. Using pure-tone audiometry as the reference, the cutoff value of the count of risk factors for predicting hearing loss was obtained by the receiver operating characteristic curve (ROC) analysis.

### Analog audiometry

In the study by Min Zhang 2019 [18], using the two tones (2 kHz and 0.5 kHz), which were selected by the decision tree analysis was applied to screen hearing loss in a community-based geriatric population. First, the better ear was tested with 42 dB/HL audio of 2 kHz twice. If there was no perception of the stimulus sound or it was only heard once, it was directly judged as moderate or above hearing loss. If it was heard twice, we further tested with the 47 dB/HL audio of 0.5 kHz twice. If heard twice, it was judged as normal or mild hearing loss. If there was no perception of the stimulus sound or it was only heard once, it was judged as moderate or above hearing loss. The other ear was tested similarly. Normal and mild hearing loss were judged as "passed", and moderate and above hearing loss were judged as "failed". Accuracy, sensitivity and specificity of a simple two-step screening procedure was 91.20%, 95.35% and 86.85%.

According to the above procedure, the study adopted two professional analog audio sources (2 kHz 42 dB/HL and 0.5 kHz 47 dB/HL), and set up simulated audiometry in the screening software. The testing environment was supposed to be in a relatively quiet environment with maximum volume of the phone.

### Screening software of ARHL

#### Design of screening software

From January to June 2020, screening software for hearing loss was developed on the WeChat platform and was written in JavaScript. The software collected the basic data of older adults via questionnaires. The three-step evaluation was as follows: assessment of risk factors, HHIE-s and analog audiometry, and output of the corresponding conclusions and suggestions.

#### Suitability evaluation of screening software

From February to May 2021,106 general practitioners from the community health centers were invited to evaluate the suitability of the procedure through an online questionnaire. The Likert scale questionnaire included the following responses: full agreement, basic agreement, general agreement, little agreement, and disagreement, corresponding to 5, 4, 3, 2 and 1 points, respectively. The contents include: basic information: age, gender, educational background, professional title and working years. Compulsory questions: ① are you willing to use the screening software for hearing loss? ② Do you think the structure of screening software is reasonable? ③ Do you think the screening software is suitable for older adults? ④ Do you think the screening software will help to raise the attention of older adults? ⑤ Do you think the screening software can assist in managing ARHL in community health service centers? ⑥ Will you use the screening software for older adults? ⑦ Do you think there are defects in the screening software? ⑧ Do you think the screening software will increase the work burden? Questions 1 to 6 are forward-scoring, while questions 7 and 8 are reverse-scoring.

#### Verification of the performance of the screening software of ARHL

From July 2020 to January 2021, 135 older adults from the community were recruited by general practitioners for pre-testing of screening software.

### Statistical analysis

EpiData 3.0 was used to input data, and SPSS 25.0 software was used for statistical analysis. The description of normal distribution data was represented by *X* ± s and compared by *T* test. The description of non-normal distribution data was represented by M (*P*25, *P*75) and compared by Mann–Whitney *U* test. Categorical data were represented by frequency and rate. The Chi-square test was used to compare non-ordinal categorical variables, and the rank sum test was used to compare ordinal categorical variables. The related factors were analyzed by Pearson, Spearman, and Logistic regression model. *P* value < 0.05 was statistically significant.

## Results

### Assessment of risk factors of ARHL

**1.1** Basic information of participants: a total of 401 older adults were enrolled in the cross-sectional study, including 182 (38.4%) males, 219 (54.6%) females, average age (71.0 ± 6.1) years. Take PTA detection as the gold indicator, The prevalence of hearing loss in males was 84.9%, while in females was 78.8%, with no statistically significant difference (*χ*^*2*^ = 2.691, *P* = 0.101). The constituent ratios of mild, moderate, moderately severe, and profound hearing loss were 48.5%, 23.4%, 7.8%, 1.3% and 0.7%, respectively. (Table 1).

**Table 1:** Basic information of participants

Variables	*n* (%)
Socio-demographic	
Age	71.0 ± 6.1
Gender	
Male	182 (45.4%)
Female	219 (54.6%)
Residential area	
Urban	231(57.6%)
Urban–rural	83(20.7%)
Rural	87(21.7%)
Whether living alone	
Living alone	30(7.5%)
Non-living alone	371(92.5%)
Marital status	
Divorced	6(1.5%)
Widowed	36(9.0%)
Married	359(89.5%)
Education level	
Primary school and below	70(17.5%)
Secondary school	288(71.8%)
Bachelor's degree or above	42(10.5%)
Lifestyle	
History of smoking	
Smoking	71(17.7%)
No-smoking	330(82.3%)
History of drinking	
Drinking	41(10.2%)
Non-drinking	360(89.9%)
Exercising habit	
Have exercising habit	209 ( 51.9%)
No exercising habit	192 ( 48.1%)
Diet	
Light diet	289 ( 72.1%)
Non-light diet	112 (27.9%)
Headphone-wearing habit	29 (7.2%)
Chronic otitis media	
Physiological	
Height(cm, *x* $\pm s$)	162.1 $\pm$ 11.1
Weight(kg, *x* $\pm s$)	64.1 $\pm$ 10.1
BMI(kg/m^2^, *x* $\pm s$)	24.45 $\pm$ 8.9
Overweight/obese	
Blood pressure	
HBP (mmHg, *x* $\pm s)$	136.4 $\pm$ 17.4
SBP (mmHg, x $\pm s$)	79.8 $\pm$ 10.1
History of chronic disease	
Hypertension	258 (64.3%)
Diabetes	123 (30.7%)
Cardiovascular disease	98 (24.4%)
Hyperlipidemia	37 (9.2%)
Hyperuricemia	68 (17.0%)
History of chronic otitis media	1 (0.2%)
Hearing loss (score > 25 dB/HL)	320 (79.8%)
HHIE-s (score > 8)	232(57.9%)

#### Logistic regression model

With the hearing loss of older adults as the dependent variable and the 22 remaining variables as the independent variable, binary logistic regression analysis was carried out (the values of the variables were as follows: gender variables were female = 1, male = 2; age and BMI were continuous variables, other independent variables and dependent variables were assigned no = 0, yes = 1). The results of univariate logistic regression analysis showed that age, BMI, overweight/obesity, history of noise exposure, non-light diet, no exercising habit, hypertension, diabetes, hyperlipidemia, cardiovascular and cerebrovascular diseases, hyperuricemia, and hypothyroidism were significant factors affecting hearing loss in older adults (*P* < 0.05).

The univariate logistic regression model showed that age, overweight, widowed/divorced, history of noise exposure, non-light diet, no exercising habit, hypertension, diabetes mellitus, cardiovascular disease, hyperuricemia, and hypothyroidism could significantly promote ARHL (Table 2).

**Table 2:** Univariate logistic regression model

Variable	*β*	SE	Wald *X*^*2*^	*P* valve	Exp(B)	95%* CI*	
Age	0.116	0.027	17.965	0.000	1.123	1.064	1.185
Sex	0.365	0.255	2.061	0.151	1.441	0.875	2.373
BMI	0.118	0.042	7.927	0.005	1.125	1.037	1.222
Overweight/obesity	0.551	0.259	4.516	0.034	1.734	1.044	2.881
Living alone	0.532	0.552	0.929	0.335	1.702	0.577	5.024
Widowed/divorced	-0.923	0.542	2.902	0.088	0.397*	0.137	1.149
Educational background of primary school and below	0.278	0.357	0.609	0.435	1.321	0.657	2.657
History of noise exposure	1.368	0.611	5.011	0.025	3.928	1.186	13.015
Family history of deafness	0.839	1.062	0.625	0.429	2.315	0.289	18.541
Non-light diet	-1.201	0.358	11.219	0.001	0.301	0.149	0.608
No exercising habit	-1.498	-0.332	20.300	0.000	0.301	0.149	0.608
Smoking	0.262	0.344	0.580	0.446	0.769	0.392	1.511
Drinking	0.656	0.494	1.760	0.185	1.927	0.731	1.538
Headset wearer	19.924	7463.606	0.000	0.998	449,668,255.2	-	-
History of chronic otitis media	19.541	40,192.970	0.000	1.000	410,198,941.3	-	-
Hypertension	1.237	0.257	23.144	0.000	3.445	2.081	5.702
Diabetes	1.794	0.412	18.936	0.000	6.001	2.680	13.484
Hyperlipidemia	0.963	0.290	11.021	0.001	2.620	1.484	4.627
Cardiovascular disease	1.273	0.393	10.508	0.001	3.571	1.654	7.708
Hyperuricemia	1.571	0.532	8.738	0.003	4.812	1.698	13.640
Hypothyroidism	2.317	1.022	5.141	0.023	10.141	1.369	75.113
History of ototoxic drug use	19.845	17,974.837	0.000	0.999	415,407,816.7	-	-

1) Overweight/obesity is defined as BMI ≥ 24.0 kg/m2; History of noise is defined as continuous operation > 1 year in an environment > 85 dB; Smoking is defined as smoking > 1 piece/day for 6 consecutive or cumulative months; Drinking is defined as drinking more than once/week, continuous or cumulative 12 months; Exercise habit is defined as 0.5 h/time and > 3 times/week; headset wearer habit is defined as wearing > 3 days/week, continuous or cumulative 12 months

2) "-" indicates that there are too few positive cases, resulting in extreme OR value, so 95% CI is not displayed

Continue incorporating the variables (*P* < 0.2) from the univariate logistic regression model into the multivariate logistic model. It was found that age growth [*OR* = 1.100, 95% *CI* (1.037, 1.166)], history of noise exposure [*OR* = 3.886, 95% *CI* (1.077, 14.022)], non-light diet [*OR* = 2.445, 95% *CI* (1.127, 5.305)], hypertension [*OR* = 1.839, 95% *CI* (1.015, 3.330)], diabetes [*OR* = 4.310, 95% CI (1.817, 10.225)], and hyperuricemia [*OR* = 3.174, 95% *CI* (1.030, 9.779)] were independent risk factors for hearing loss in older adults (*P* < 0.05) (Table 3).

**Table 3:** Multivariate logistic regression model

Variable	*β*	SE	Wald *X*^*2*^	*P* valve	Exp(B)	95% CI	
Age	0.104	0.031	11.221	0.001	1.100	1.037	1.166
Sex	− 0.056	0.322	0.030	0.863	0.946	0.503	1.779
BMI	0.059	0.075	0.609	0.435	1.060	0.915	1.228
Overweight/obesity	− 0.081	0.468	0.030	0.836	0.922	0.368	2.310
Widowed/divorced	0.148	0.712	0.043	0.836	1.159	0.287	4.681
History of noise	1.357	0.655	4.298	0.038	3.886	1.077	14.022
Non-light diet	0.894	0.395	5.114	0.024	2.445	1..127	5.305
No exercise habit	− 0.385	-0.403	0.915	0.339	0.680	0.309	1.498
Drinking	0.741	0.635	1.359	0.244	2.098	0.604	7.287
Hypertension	0.609	0.303	4.038	0.044	1.839	1.015	3.330
Diabetes	1.461	0.441	10.985	0.001	4.310	1.817	10.225
Hyperlipidemia	0.246	0.355	0.479	0.489	1.278	0.637	2.564
Cardiovascular disease	0.362	0.463	0.613	0.434	1.437	0.580	3.558
Hyperuricemia	1.155	0.574	4.045	0.044	3.174	1.030	9.779
Hypothyroidism	1.645	1.066	2.379	0.123	5.181	0.641	41.897

#### Hierarchical evaluation system of risk factors of ARHL

According to the results of the logistic regression model, variables as non-light diet, hypertension, diabetes, hyperlipidemia, and history of noise exposure were defined as risk factor of age-related hearing loss. The univariate analysis tipped the overweight/obesity, living alone, divorce/widowhood, non-exercise habit, family history of deafness, smoking history, drinking history, hypothyroidism, hyperuricemia history of wearing headphones, history of ototoxic drug was with statistical significance. In combination with the mention rate of risk factors in literature and expert recommendations [21, 22, 27, 29] the above variables were also defined as risk factors. Population surveys [21, 22, 27, 29] suggested that the incidence of hearing loss in male was higher than female. In the study, there was sex difference in the incidence rate, with no statistical significance. Considering possible selection bias, male was included in the risk factors. Participants of the study were older adults, so age was not included in the risk factors. Finally, 18 risk factors were included in the list.

**Fig. 1:**
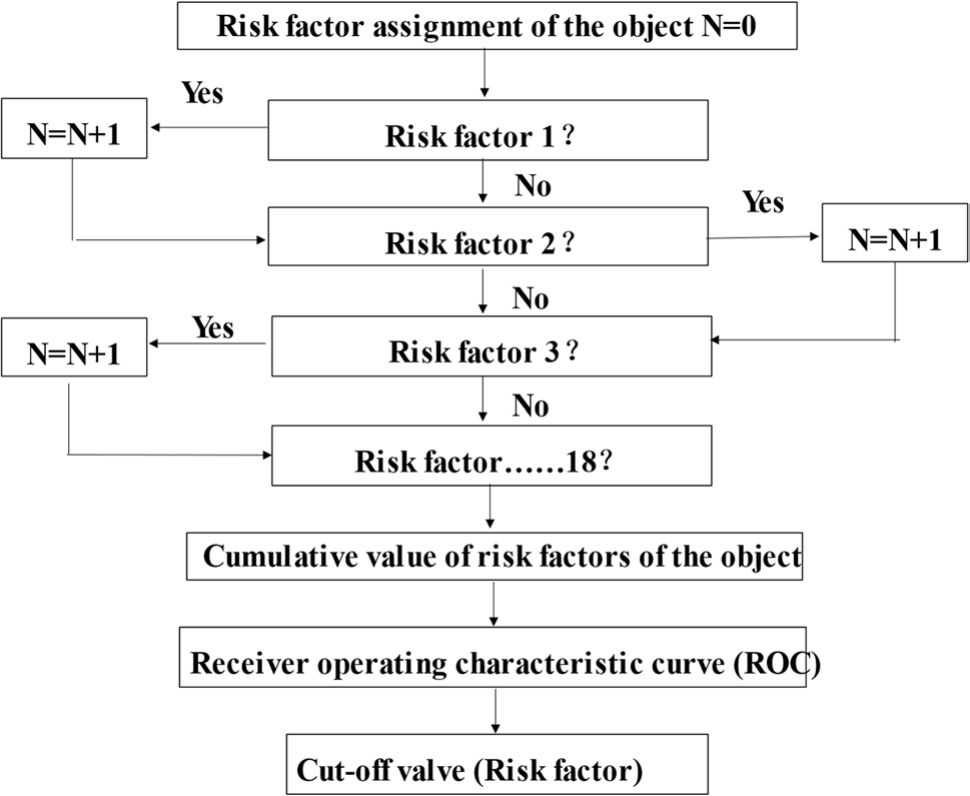
Hierarchical assessment system of risk factors of age-related hearing loss

The conditional logic equation was used to calculate the cumulative score of risk factors. Receiver operating characteristic curve (ROC) analysis was conducted to assess the predictive value of the cumulative score of risk factors on hearing loss in older adults was obtained. The results showed the AUC (area under the ROC curve) was 0.777 [95% *CI* (0.721, 0.833)], the maximum Youden index was 0.534, and the optimal cutoff value was 3.5. The sensitivity and specificity were 70.9% and 75.3%, respectively. The threshold of the cumulative score of risk factors was 4, and used to classify older adults into low-risk (< 4) and high-risk (≥ 4) groups. (Fig. 1).

### Screening software of ARHL

#### Basic structure

The screening software was written in JavaScript language. The structure included three layers: foundation, display, and processing. The software provides dual-port management. Older adults used the WeChat platform terminal, and general practitioners used computer terminals. Older adults in the community could conduct dynamic self-assessments without geographical, time and space restrictions. The screening data could be updated in real time and synchronously to ensure that general practitioners could track and follow-up from time to time by the computer terminal.

#### Suitability setting

1. Each subject's WeChat login has a unique ID code, and the assessment could be repeated, suggesting it is suitable as a follow-up tool.
2. The program interface uses large font and simplified expression, suitable for older adults.
3. The program is easy to operate and does not occupy the mobile phone's storage space.
4. All questions were single-choice ones. The language was concise with no ambiguity, convenient for older adults to operate and provided accurate feedback for data collection.
5. The two-step analog audiometry adopts the standardized analog audio recognized in the industry and uses the measurement of maximizing the volume of the external amplifier. The requirements for listening equipment and listening environment are relatively low.

#### Five sections

1) Assessment of risk factors:

A cumulative score of risk factors was obtained from the questionnaire and divided testers into high-risk or low-risk groups.

2) HHIE-s:

Testers were divided into normal hearing and hearing loss groups.

3) Two-step analog audiometry:

The analog audio was played through the mobile phone terminal using the two tones (2 kHz 42 dB/HL and 0.5 kHz 47 dB/HL) selected by decision tree analysis. Normal and mild hearing loss were judged as "passed", and moderate and above hearing loss were judged as "failed" [18].

4) Conclusion

If the risk factor is assessed as low risk, the HHIE-s score is 0–8, and the two-step pure tone audiometry result was normal, the following suggestion would be given: conduct a hearing loss screening software self-assessment every 6 months.

If the risk factor is assessed as high risk, the score of HHIE-s was ≥ 10, and the two-step pure tone audiometry result was abnormal, the following suggestion would be given: conduct a hearing loss screening software self-assessment every 3 months.

5) Health education:

Participants received health education to prevent hearing loss and were regularly updated on educational programs on hearing loss.

#### Suitability evaluation of screening software of ARHL

1) Basic information:

106 general practitioners from community health centers had participated in the questionnaire, and the basic information as follows: male 30 (28.7%), average age 38.35 ± 7.98, graduate degree or above 10(9.4%), over 10 of working years 71 (57.7%), intermediate and above professional titles 81 (75.5%). The response rate of participants to the questionnaire was 100% (supplemental Table 1).

2) Questionnaire of suitability evaluation:

The score distribution suggests that general practitioners approved the structure, convenience, and value of the hearing loss screening software (Table 4).

**Table 4:** Questionnaire for suitability evaluation of screening software of ARHL

	Score	Select percentage n (%)
Are you willing to use the screening software for age-related hearing loss?	4	15 (14.2)
	5	90 (857)
Do you think the structure of screening software is reasonable?	3	1 (1.0)
	4	19 (18.1)
	5	85 (81.0)
Do you think the screening software is suitable for older adults?	4	29 (27.6)
	5	76 (72.4)
Do you think the hearing loss screening software will help raise the attention of older adults?	3	9 (8.6)
	4	24 (22.9)
	5	72 (68.6)
Do you think the hearing loss screening software can assist in the management of age-related hearing loss?	3	8 (7.6)
	4	26 (24.8)
	5	71 (67.7)
Will you use the hearing loss screening software for older adults?	3	10 (9.5)
	4	33 (31.4)
	5	62 (59.0)
Do you think there are defects in the screening software? *	3	17 (16.2)
	4	29 (27.7)
	5	59 (56.2)
Do you think using screening software will increase the work burden? *	3	15 (14.3)
	4	28 (26.7)
	5	62 (59.0)

^*^Indicates a negative score

#### Verification of the performance of the screening software of ARHL

All 135 older adults in the community were randomly recruited by the general practitioner through the WeChat platform to participate in software testing and pure tone audiometry. Finally, 109 persons with a mean age of 69.17 ± 7.3 years and exhibiting male predominance (*n* = 72, 66.1%) completed the screening software test and pure tone audiometry, with a participation rate of 80.7%.

Correlation analysis showed that the spearman coefficient between PTA and the results of HHIE-s, the cumulative score of risk factors, and two-step analog audiometry was 0.704, 0.672, and 0.710, respectively (*P* < 0.001). Taking PTA as the reference, the conclusion of screening software was evaluated from three aspects: authenticity, reliability, and predictive value. The sensitivity, specificity, positive predictive value, and negative predictive value were 100%, 65.5%, 71.8%, and 100.0%, respectively. The Kappa index was 0.6.

26 elderly persons did not complete the screening software evaluation for reasons listed as follows (selection ratio from high to low): "Test is of little significance" 14 (54.8%), "No further willingness to test" 10 (38.7%), "Test procedure is too troublesome" 4 (15.4%), "Worried about personal privacy disclosure" 4 (15.4%), "Too many test sections" 3 (11.5%), "Some questions are not understood" 2 (7.7%), "Test environment is not suitable" 1 (3.8%), "Unexpected signal interruption during test" 1 (3.8%) (supplemental Table 2).

## Discussion

### Hierarchical assessment system of risk factors of ARHL

Based on the theoretical model of health risk factors, risk factors were divided into four levels (biological factors, environmental factors, disease factors, behavior, and lifestyle factors). Logistic regression analysis was applied to the data of 401 elderly persons, and the interaction effects were assessed. The risk factors were: (1) biological factors: gender, family history of deafness, (2) environmental factors: living alone, marital status, noise exposure history, (3) disease factors: high blood pressure, diabetes, hyperlipidemia, cardiovascular disease, hypothyroidism, hyperuricemia, ototoxic drug use history, and (4) behavior and lifestyle factors: being overweight, diet, exercise, smoking history, drinking history, headset wearing history.

Although the current literature suggests that regional distribution and education level are risk factors for hearing loss, this finding was not observed in our study. Accordingly, these two factors were not included. Variables mentioned in the literature, including gender, education background, family history of deafness, smoking, drinking, headset wearer habit, history of chronic otitis media, and history of ototoxic drug use, did not exhibit statistical significance. These findings may be attributed to the following reasons. Since convenience sampling was adopted, there may be a bias in the included elderly persons, lacking certain representativeness. Besides, variables such as smoking and drinking had no significant difference, which may be attributed to the imbalanced gender ratio. Moreover, the actual number of cases was too small for some parameters (headset wearer, history of chronic otitis media and history of ototoxic drug use), leading to inaccurate results.

Compared with diabetes, hypertension, and other chronic diseases, ARHL has a high prevalence and brings many adverse events and a heavy disease burden. Although primary prevention of ARHL is challenging, it is widely thought that 72.2% (13/18) of these factors are preventable, controllable, delayable, and improvable.

#### Development of hearing loss screening software based on the WeChat platform

The US Preventive Services Task Force summarized and analyzed 20 screening tests, including 4 from primary health care and 16 from residents. The results showed that no hearing screening tool has sufficient reliability and positive and negative predictive values [7]. Although ARHL does not belong to the chronic non-communicable diseases (NCD) category, its incidence is related to various behavioral factors. Hearing loss can cause serious damage to health, which is often irreversible. General practitioners play an important role in preventing and treating ARHL, especially in older adults. However, it remains unclear how to establish an effective and economic screening model, emphasizing the need for further research.

The advantages of the hearing test software include simulated multi-frequency pure-tone audiometry and professional test results, including listening age, listening bulletin, audiogram, and language area. Finally, relatively complete conclusions can be obtained. However, it should be borne in mind that the development team consists mostly of hearing experts, and the test focuses more on specialized knowledge of hearing. Given that the operation and conclusion are too complex, subjects cannot understand them independently. Moreover, this approach does not consider high-risk factors, self-management ability, diagnosis, and treatment compliance. The methods can realize the online service function of specialist doctors, but it cannot realize the closed-loop management of hearing loss screening, and cannot promote the formation of self-health management beliefs of high-risk groups of hearing loss.

In the study, the hierarchical assessment system of risk factors, HHIE-s, two-step pure analog audiometry and the WeChat platform were optimized and integrated to construct the self-assessment software for screening of ARHL, yielding the following advantages and effects. For the operation section, the interface is friendly and in line with the usage habits of older adults. WeChat programs do not occupy the storage space of mobile phones and have low requirements for mobile phone configuration. At present, intelligent elderly mobile phones can meet the test requirements. For the function section, the WeChat platform terminal is required for elderly residents and a computer terminal for general practitioners. Older adults can be tested from time to time, regardless of time, space, and geographical restrictions. General practitioners can inspect the test data at predefined intervals, and the ID number can be dynamically compared. To implement self-health management, the software realizes real-time data transmission, early warning, management, whole process supervision and closed-loop management process. For the screening methods, the software was set as a three-layer framework: (1) the conditional logic equation was adopted to assign and count the risk factors of the included individuals. Through the boundary value, the hierarchical assessment of risk factors was obtained; 2) the HHIE-s scale was adopted to evaluate the hearing loss and speech communication situation by simulating daily life scenes, reflecting the extent of hearing loss and communication barriers of the elderly, and forming a multidimensional and multi-level evaluation; and 3) the program adopts the two-step pure tone audiometry based on the decision tree intelligent algorithm, which breaked through the bottleneck that the previous clinical standard pure tone range audiometry cannot take into account the accuracy, time cost and economic cost, and being difficult to carry out effective development among the large population in the coverage area of the grass-roots community health center ^16^, the method could realize the sensitivity of 95.4% for the recognition of moderate and above deafness within 30 s. The analog audio signals used were recognized by the industry, and subjects could obtain their own hearing loss measurement results more quickly and easily. According to the results of the above three steps, the general practitioner can monitor in real time on the PC terminal, find the high-risk population of ARHL in time, and implement effective screening and timely intervention. The software sets up regular health education content and real-time updated special education content for hearing loss, which can promote the perceived susceptibility, perceived severity, perceived benefits and cue to action of the elderly to hearing loss based on health belief mode.

### Suitability evaluation of screening software of ARHL

106 general practitioners from the community health service centers were invited to evaluate the suitability of screening software for hearing loss through an online questionnaire. The majority had a bachelor's degree or above (96.2%), worked for more than 10 years (57.6%), and had intermediate or above professional titles (75.5%). The questionnaire adopted a 5-point Likert subscale with 8 single-choice questions, focusing on the evaluation of using intention, convenience, framework structure, and significance. For question 1: "Are you willing to use the screening software of ARHL?", 85.8% chose full agreement and 14.4% chose basic agreement. Question 2: "Do you think the structure of screening software is reasonable?", 99.1% of general practitioners basically or generally agreed. Question 4: "Do you think the use of hearing loss screening software will help to raise the attention of older adults?" 92.5% of general practitioners agreed with this view. Therefore, from the perspective of grass-roots general practitioners, the promotion and use of elderly hearing loss screening software are feasible. Importantly, our screening software, based on the intelligent medical terminal, enables online data transmission in real time, which provides the platform to help hearing loss screening among the older adults.

### Verification of screening software of ARHL

135 older adults were selected for the pre-test. 109 participants were finally included, with a participation rate of 80.7%. The correlation analysis showed that the HHIE-s, assessment of risk factors and two-step analog audiometry were moderately correlated with PTA. Moreover, the sensitivity, specificity, positive predictive value, and negative predictive value were 100%, 65.5%, 71.8%, and 100.0%, respectively.

26 participants did not complete the pre-test for the following reasons: "the test is of little significance" (54.8%), "no further willingness to test" (38.7%), "test procedure is too troublesome" (15.4%), "worried about personal privacy disclosure" (15.4%), and "too many screening items" (11.5%).

The main reasons for not completing the screening software evaluation were low willingness and lack of health knowledge. With the help of general practitioners, such as strengthening health education on hearing loss, fostering the use of screening software, and seeking the assistance of family members, we can improve the awareness of older adults on hearing loss and the acceptance of screening.

## Conclusion

The screening software for age-related hearing loss documented in this study enables early screening and intervention through real-time data transmission to achieve self-health management among elderly residents. At the same time, from the perspective of primary care and general practitioners, our screening software meet the need for large-scale hearing detection in community-dwelling older adults, provides a new approach to hearing loss control which can be easily implemented.

## Declaration

## Conflict of interest

There are no conflicts of interest to declare.

## Ethics statement

Ethics approval and consent to participate Ethics approval by the Academic Ethics Committee of Shanghai East Hospital (wf2021094) was acquired prior to the current study, which did not involve any ethical issues.

### Supplementary Information

Below is the link to the electronic supplementary material.

Supplementary file1 (DOCX 16 KB)

Supplementary file2 (DOCX 15 KB)

## Data Availability

Data description: cross section data，no missing data in the dataset. Data usage: Data can be referenced by similar studies. By providing this data availability statement, we aim to promote transparency and reproducibility in scientific research. We believe that making this data available will contribute to a better understanding of the research topic and facilitate further investigations.
