# Supplementary material for: Development and validation of the screening tool for age-related hearing loss in the community based on the information platform
Source: Eur Arch Otorhinolaryngol. 2024 Jan 11;281(6):2893–903. doi: 10.1007/s00405-023-08389-9 (PMC11065916; doi:10.1007/s00405-023-08389-9)
Supplement: Supplementary file 1 — Supplementary file1 (DOCX 16 KB) [file 405_2023_8389_MOESM1_ESM.docx]

Supplemental Table 1 Basic Information of the general practitioners

| Items | Number (%) |
| --- | --- |
| Sex |  |
| male | 30 (28.7) |
| female | 76 (71.7) |
| Age (year) |  |
| 20-30  31-40  41-50  >50 | 16 (15.1)  42 (39.6)  43 (40.6)  5 (4.7%) |
| Education  College  Undergraduate  graduate or above | 4 (3.8%)  92 (86.8)  10（9.4） |
| Working years  1-5  6-10  11-15  > 15 | 26 (24.5)  19 (17.9)  16 (15.2)  45 (42.5) |
| Professional title  Junior  Intermediate  senior | 24 (24.5)  75 (70.8)  6 (5.7) |
