# Supplementary material for: Development and validation of the screening tool for age-related hearing loss in the community based on the information platform
Source: Eur Arch Otorhinolaryngol. 2024 Jan 11;281(6):2893–903. doi: 10.1007/s00405-023-08389-9 (PMC11065916; doi:10.1007/s00405-023-08389-9)
Supplement: Supplementary file 2 — Supplementary file2 (DOCX 15 KB) [file 405_2023_8389_MOESM2_ESM.docx]

Supplemental Table 2 Reasons why older adults did not complete the screening software evaluation

| Reason | n（%） |
| --- | --- |
| Test is of little significance | 14 (54.8) |
| No further willingness to test | 10 (38.7) |
| Test procedure is too troublesome | 4 (15.4) |
| Worried about personal privacy disclosure | 4 (15.4) |
| Too many test sections | 3 (11.5) |
| Some questions are not understood | 2 (7.7) |
| Test environment is not suitable | 1 (3.8) |
| Unexpected signal interruption during test | 1 (3.8) |
